# SECONDARY SCHOOL CURRICULAR RIGOR AND COGNITIVE FUNCTION AT AGE 81

**DOI:** 10.1093/geroni/igad104.0003

**Published:** 2023-12-21

**Authors:** Sara Moorman

**Affiliations:** Boston College, Boston, Massachusetts, United States

## Abstract

A current goal is to identify how and for whom years of educational attainment translate into high cognitive function in later life. Recent work has found effects of school quality factors such as teacher experience and racial integration of schools. In this project, I examine secondary school curriculum. Not only is there variation in curriculum across school districts, but also adolescents within a single secondary school select different courses. Curricular opportunities and selections may change the developing brain in lasting ways, and/or may place the student on a path towards future academic and career opportunities that shape risk and resilience. I hypothesize that a rigorous secondary school curriculum positions students to attend college, which in turn promotes lifelong cognitive health. I tested this hypothesis by estimating multilevel structural equation models in data from the Wisconsin Longitudinal Study (WLS), which follows a randomly-selected one-third of all students who graduated from Wisconsin high schools in 1957 (N = 10,317). I created a latent measure of curricular rigor out of participants’ highest secondary school math course and number of semesters of English, science, and foreign language. In 2020 (most participants were age 81), participants completed a phone-based cognitive screening (Telephone Interview for Cognitive Status-Modified; TICS-m). Net of school-level factors, parental socioeconomic status, and academic achievement, higher curricular rigor predicted higher cognitive function at age 81. Educational attainment significantly mediated this effect, and a significant direct effect also remained. Both educational attainment and curricular rigor are desirable targets for policy interventions.

